# Advancing Genetically Encoded Lysine (GEK) Chemistry: From Precision Modulation to Therapeutic Innovation

**DOI:** 10.1021/acs.accounts.5c00401

**Published:** 2025-09-26

**Authors:** Guoqing Jin, Yifan Shi, Shivangi Sharma, Wenshe Ray Liu

**Affiliations:** † Texas A&M Drug Discovery Center and Department of Chemistry, College of Arts and Sciences, 14736 Texas A&M University, College Station, Texas 77843, United States; ‡ Institute of Biosciences and Technology and Department of Translational Medical Sciences, College of Medicine, Texas A&M University, Houston, Texas 77030, United States; § Department of Biochemistry and Biophysics, College of Agriculture and Life Sciences, Texas A&M University, College Station, Texas 77843, United States; ∥ Department of Cell Biology and Genetics, College of Medicine, Texas A&M University, College Station, Texas 77843, United States; ⊥ Department of Pharmaceutical Sciences, Lerma Rangel College of Pharmacy, Texas A&M University, College Station, Texas 77843, United States

## Abstract

Genetically encoded lysine (GEK) chemistry has transformed protein engineering by enabling precise and site-specific modifications, which expand lysine’s functional landscape beyond its native post-translational modifications (PTMs). Our work has systematically advanced GEK chemistry by developing engineered pyrrolysyl-tRNA synthetase (PylRS) variants that efficiently incorporate diverse lysine (Lys) derivatives with tailored chemical reactivity. By integrating bioorthogonal handles, acyl and electrophilic warheads, photo-cross-linking groups, and PTM mimics, we have established a set of powerful toolkits for protein labeling, functional studies, and Lys-directed drug design. These advances provide precise control over protein structure and function, facilitating the study of epigenetic modifications, enzyme–substrate interactions, and Lys-guided inhibitor development. As GEK chemistry continues to evolve, its integration with structural/synthetic biology and therapeutic innovation will further expand its impact, unlocking new frontiers in chemical biology and precision therapeutics.

## Graphical abstract

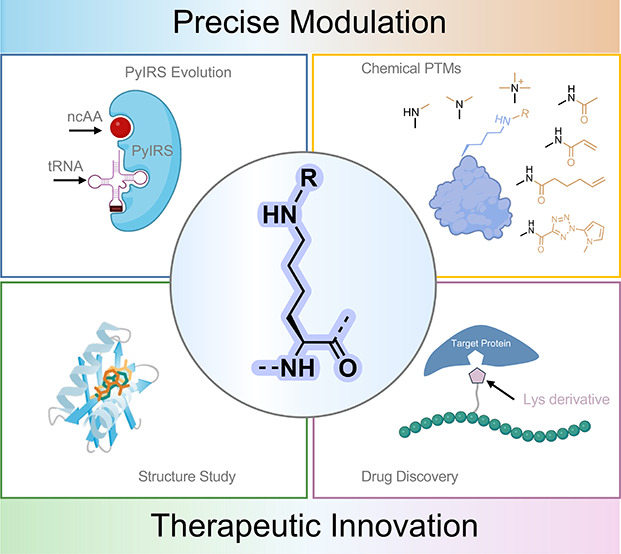

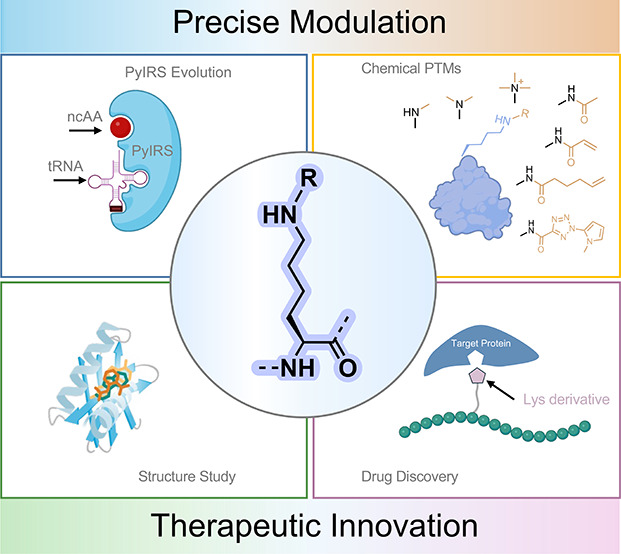

## Key References

-

Wang, Z. A. ; Zeng, Y. ; Kurra, Y. ; Wang, X. ; Tharp, J. M. ; Vatansever, E. C. ; Hsu, W. W. ; Dai, S. ; Fang, X. ; Liu, W. R. A Genetically Encoded Allysine for the Synthesis of Proteins with Site-Specific Lysine Dimethylation. Angew. Chem., Int. Ed. 2017, 56, 212–216 10.1002/anie.201609452PMC520689327910233.^1^ A method that allows expedient synthesis of proteins with site-specific lysine dimethylation has been successfully demonstrated.
-

Wang, W. W. ; Angulo-Ibanez, M. ; Lyu, J. ; Kurra, Y. ; Tong, Z. ; Wu, B. ; Zhang, L. ; Sharma, V. ; Zhou, J. ; Lin, H. ; Gao, Y. Q. ; Li, W. ; Chua, K. F. ; Liu, W. R. A Click Chemistry Approach Reveals the Chromatin-Dependent Histone H3K36 Deacylase Nature of SIRT7. J. Am. Chem. Soc. 2019, 141, 2462–2473 30653310 10.1021/jacs.8b12083PMC6812484.^2^ The study identifies H3K36 as a physiologic deacetylation target of SIRT7 using genetically encoded acyl-nucleosomes and click-labeling assays.
-

Tharp, J. M. ; Hampton, J. T. ; Reed, C. A. ; Ehnbom, A. ; Chen, P.-H. C. ; Morse, J. S. ; Kurra, Y. ; Pérez, L. M. ; Xu, S. ; Liu, W. R. An Amber Obligate Active Site-Directed Ligand Evolution Technique for Phage Display. Nat. Commun. 2020, 11, 1392 32170178 10.1038/s41467-020-15057-7PMC7070036.^3^ The study overcomes amber suppression bias in phage libraries by using superinfection immunity to enrich ncAA-containing clones, enabling active-site directed evolution of SIRT2-binding peptide inhibitors.
-

Chen, P.-H. C. ; Guo, X. S. ; Zhang, H. E. ; Dubey, G. K. ; Geng, Z. Z. ; Fierke, C. A. ; Xu, S. ; Hampton, J. T. ; Liu, W. R. Leveraging a Phage-Encoded Noncanonical Amino Acid: A Novel Pathway to Potent and Selective Epigenetic Reader Protein Inhibitors. ACS Cent. Sci. 2024, 10, 782–792 38680566 10.1021/acscentsci.3c01419PMC11046469.^4^ The study introduces a phage-assisted active-site directed evolution of peptide inhibitors of the ENL YEATS domain for leukemia therapy.

## 1 Introduction

Lysine (Lys) is a biologically indispensable amino acid, playing a central role in protein structure and function and undergoing a diverse array of post-translational modifications (PTMs) that regulate many aspects of cellular physiology in multicellular organisms.^5–7^ The unique ε-amino group of Lys confers high nucleophilicity and chemical versatility, making it a primary site for a diverse array of PTMs, including, but not limited to acetylation,^5,8^ methylation,^1,6,9^ and ubiquitination.^10^ These modifications regulate fundamental biological processes, such as chromatin remodeling, transcriptional regulation, signal transduction, and protein homeostasis, influencing key cellular outcomes such as differentiation, metabolism, and immune responses. Given their significant roles in biology, Lys modifications are tightly regulated, and their dysregulation is linked to numerous diseases, including cancer,^11,12^ neurodegeneration,^13^ protein degradation,^14^ and infectious diseases.^15^

Among Lys modifications, acetylation^5^ and methylation^6,7^ have received particular attention due to their pivotal roles in epigenetic regulation. Lys acetylation, which is carefully balanced by histone acetyltransferases (HATs) and histone deacetylases (HDACs), modulates gene expression by altering chromatin accessibility. Meanwhile, Lys methylation, which is catalyzed by histone lysine methyltransferases and reversed by histone lysine demethylases, provides a different layer of epigenetic controls and influences transcriptional activation or repression depending on the specific methylation state and/or Lys site. In addition, Lys modifications occur broadly on non-histone proteins as well, such as p53, RB1, and STAT3, affecting their stability, localization, and functions.^16^ The biological significance of Lys PTMs highlights the need for precision tools to study, manipulate, and therapeutically target Lys-dependent processes.

Traditional approaches for investigating Lys PTMs have relied on enzymatic methods, such as using recombinantly expressed modifying enzymes or generating Lys mutants to mimic PTM states.^6^ However, these approaches suffer from limitations, including substrate promiscuity for most histone modifying enzymes, incomplete modifications, and undesired site-/stereoselectivity. A major challenge remains in developing methods that allow for the site-specific precise incorporation of chemically diverse Lys derivatives in a biologically relevant manner. To address this challenge, our lab has been long focused on developing genetically encoded lysine (GEK) chemistry, enabling the site-specific incorporation of Lys analogs with tailored chemical functionalities. By engineering the pyrrolysyl-tRNA synthetase (PylRS)/tRNA^Pyl^ system, we have expanded the Lys chemical space to include bioorthogonal handles, electrophilic warheads, acylated Lys mimics, and photo-cross-linking groups. These advances allow for precise Lys modifications that are orthogonal to native biological processes, opening new avenues for engineering proteins, cells, and bacterial viruses for various purposes, including the study of basic biological processes and the development of therapeutics.

In this Account, we present our laboratory’s advances in GEK chemistry since 2007 by focusing on its applications in precision protein engineering, structural and mechanistic studies of Lys PTMs, and active GEK-assisted drug discovery. We first discuss the evolution of PylRS for enhanced incorporation of lysine-based noncanonical amino acids (Lys-ncAAs), which has enabled efficient genetic encoding of Lys-ncAAs with diverse chemical functionalities, including moieties for bioorthogonal chemistry reactions. Next, we highlight how these Lys-ncAAs have provided new structural and mechanistic insights into Lys PTMs, revealing key interactions in epigenetic regulation, enzyme–substrate recognition, and chromatin dynamics. Furthermore, we show how Lys-ncAAs have facilitated the development of highly selective inhibitors of SIRT2, ENL, HDAC8, or ZNRF3. By integrating genetic code expansion techniques with bioorthogonal chemistry, we have established a set of promising platforms for precise Lys PTM or analogue installation on proteins, extending the functional scope of Lys beyond its natural PTMs. These advances not only enhance our ability to probe Lys and its PTM functions but also pave the way for next-generation therapeutic development by targeting Lys-dependent disease processes such as cancer, neurodegeneration, and metabolic disorders.

## 2 Evolution of PylRS for Genetic Incorporation of Noncanonical Amino Acids (ncAAs)

A critical challenge in biochemistry is the development of methods to precisely modify protein structures for functional investigations and potential applications. By going beyond the 20 canonical amino acids, ncAAs allow us to introduce new physical/chemical properties, such as fluorescence, photosensitivity, chemical reactivity, and even selective ligand engagement.^17,18^ Initially, artificial systems composed of an engineered aminoacyl-tRNA synthetase (AARS)–amber suppressing tRNA pair were used to incorporate ncAAs at the amber codon (UAG) in living cells.^18^ However, it was later discovered that a natural system already exists for the incorporation of pyrrolysine (Pyl), the 22nd proteinogenic amino acid but a ncAA, at the UAG. This process is facilitated by a unique AARS called PylRS and tRNA^Pyl^ that is an amber suppressor with a CUA anticodon.^17,19^ PylRS stands out from most AARSs due to its broad substrate flexibility for the *N* ^ε^-acyl group on the substrate side chain, low specificity for the α-amine group, and no direct enzymatic interactions with the tRNA^Pyl^ anticodon.^20,21^ These unique features render PylRS highly adaptable for genetic engineering, allowing the incorporation of over 200 ncAAs into proteins that are genetically encoded at the UAG codon.^22,23^ The system can also be reprogrammed to reassign other codons,^24,25^ such as ochre (UAA) or opal (UGA), four-base codons like AGGA, and even a sense codon such as AGG,^26^ to encode ncAAs, leading to a greatly expanded genetic code system (Figure 1A).^27^ Lys-ncAAs are particularly valuable due to the various roles of Lys PTMs and their involvement in numerous signaling pathways.^28^ Leveraging the versatility of PylRS, we have identified different engineered PylRS mutants for the genetic incorporation of Lys-ncAAs into proteins including incorporating multiple different ncAAs in the same protein.

**Figure 1:**
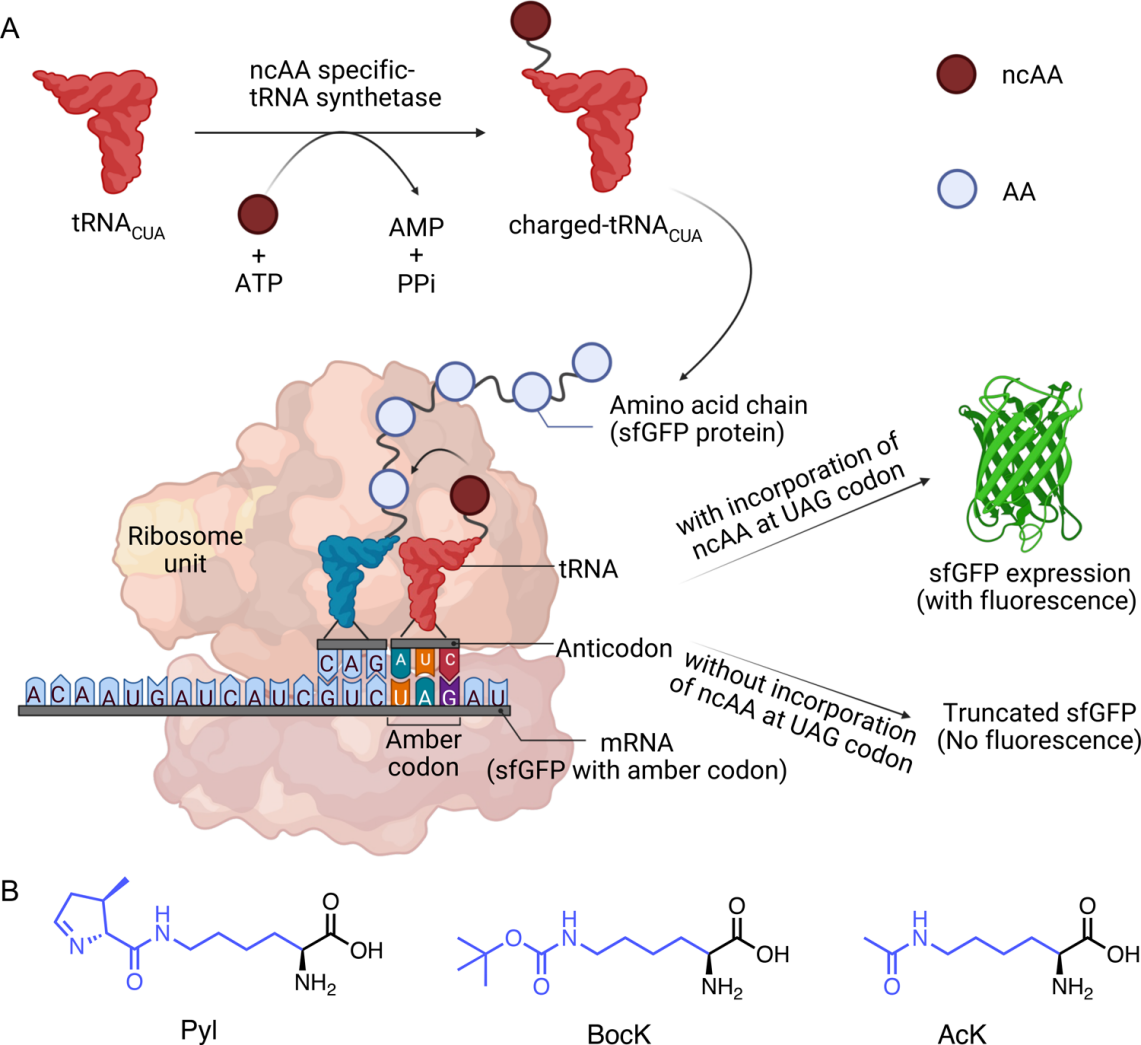
(A) Schematic diagram of incorporation of a ncAA into sfGFP via amber suppression. A ncAA-charged tRNA pairs with the CUA anticodon, enabling full-length fluorescent protein expression; absence of the ncAA leads to truncation. (B) Representative structures of ncAAs (Pyl, BocK, AcK) commonly used in this system.

### 2.1 Suppression of Nonsense Codons

Genetic code is designed in a way that only a particular amino acid can be incorporated at a specific codon.^17,18^ This precise codon usage helps ensure the correct incorporation of the corresponding amino acid during the process of translation. Similarly, in an expanded genetic code system, precise codon usage becomes critical when assigning any ncAA to a unique nonsense codon such as UAG, UAA, or UGA, or a four-base codon such as AGGA without interfering with translation of native proteins. However, there are always background suppression concerns due to partial recognition of a specific codon by canonical tRNAs with near-cognate anticodons.^29^ One of our studies investigated near-cognate suppression at amber, opal, and quadruplet codons in common *Escherichia coli* (*E. coli*) strains, and it was concluded that the PylRS/tRNA^Pyl^ orthogonal pair is unable to fully eliminate the incorporation of canonical amino acids, leading to a minimal level of contamination in the expressed proteins.^27^ One needs to be aware that this arises from the intrinsic nature of the translation system and take this aspect into consideration during the use of genetic code expansion techniques.

Suppression of nonsense (UAG, UGA, UAA) and sense (AGG) codons was further investigated in *E. coli* BL21 (DE3) strain by using *Methanosarcina mazei* PylRS (*Mm*PylRS) and its corresponding tRNA^Pyl^ to incorporate *N* ^ε^-(*tert*-butoxycarbonyl)-l-lysine (BocK) (Figure 1B). Among the tRNA^Pyl^ variants, tRNA^Pyl^_CUA_ demonstrated the highest efficiency for delivering BocK at its corresponding codon. However, tRNA^Pyl^_UUA_, while orthogonal in *E. coli*, did not significantly suppress the amber codon despite having a near-cognate anticodon. This limited cross-reactivity enables the use of different AARS pairs to incorporate two different ncAAs at UAG and UAA, respectively. Incorporation of BocK in response to UGA by PylRS/tRNA^Pyl^_UCA_ is accompanied by significant Trp incorporation due to near-cognate suppression of UGA by Trp-tRNA^Trp^_CCA_. The PylRS/tRNA^Pyl^_CCU_ pair, however, was not effective in delivering BocK at the AGG codon, suggesting that further optimization of the tRNA^Pyl^_CCU_ sequence and structure may be needed to improve its efficiency and reduce Arg incorporation at the AGG codon.^30^

### 2.2 Exploration of Multiplexed Incorporation

Two widely used strains of PylRS from archaebacteria *Methanosarcina mazei* and *Methanosarcina barkeri* share 83% sequence identity.^31^ For both strains, the full-length enzyme consists of two domains: the C-terminal catalytic domain that is responsible for catalyzing the aminoacylation of tRNA^Pyl^ and the N-terminal domain which enhances tRNA^Pyl^ binding through direct interaction but does not directly participate in aminoacylation (Figure 2A). However, the high insolubility of the N-terminal domain often causes the full-length enzyme to aggregate or causes cleavage from the C-terminal domain, rendering it inactive for tRNA charging.^32–34^ Most engineered ncAA-specific PylRS mutants have mutations in the C-terminal domain (Figure 2B), and we proposed that introducing mutations in the N-terminal domain could enhance tRNA binding affinity, thereby improving tRNA aminoacylation and boosting the incorporation efficiency of ncAAs (Figure 2C). To test this, we screened a randomized N-terminal library of *Mm*PylRS using BocK, a non-native substrate for wild-type *Mm*PylRS. The screening identified the R19H/H29R/T122S mutation combination, which significantly improved ncAA incorporation efficiency.^35^ It was further confirmed that these mutations could be transferred to other ncAA-specific *Mm*PylRS mutants, enhancing their efficiency for incorporating specific ncAAs.^36,37^ To improve full-length *Mm*PylRS stability and ncAA incorporation efficiency, a P188G mutation was introduced to suppress cleavage of the N-terminal domain that was observed upon coexpression with tRNA^Pyl^ variants.^38^

**Figure 2:**
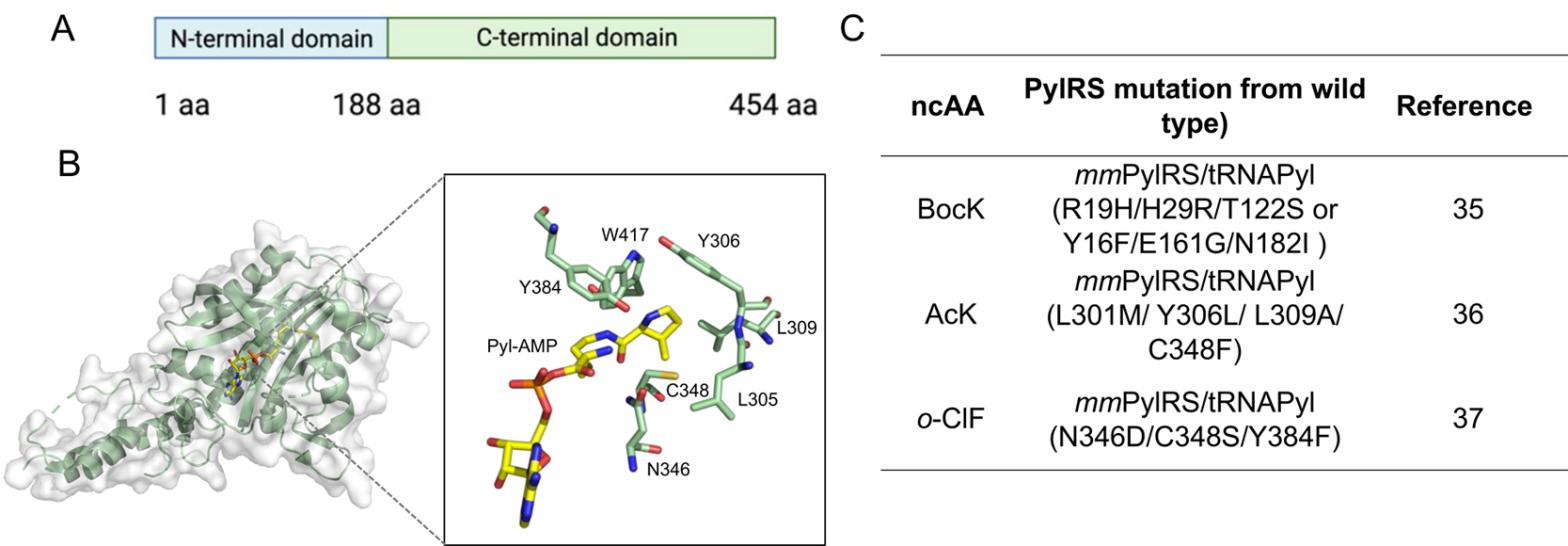
(A) Two domains of *Mm*PylRS. (B) Structure of the catalytic core of *Mm*PylRS with Pyl-AMP bound at the active site (PDB entry: 2ZIM). (C) The table of PylRS mutants for Lys-ncAA incorporation.^35–37^

Many proteins have additional chemical functionalities provided by PTMs, leading to expanded chemical diversity and increased complexity of proteomes.^39^ Expanding to multiplexed incorporation enables the installation of multiple PTMs for modeling complex biological states, but the incorporation of multiple ncAAs is not well established. By targeting the UAG and UAA codons, we successfully incorporated two different ncAAs into sfGFP. In one approach, the *Mm*PylRS–tRNA^Pyl^_UAA_ pair was used to introduce BocK at the UAA codon, while the evolved *Methanococcus jannaschii* tyrosyl-tRNA synthetase (*Mj*TyrRS)–MjtRNA^Tyr^_CUA_ pair mediated the incorporation of *p*-azidophenylalanine (AzF) at the UAG codon in GFP1TAG149TAA.^40^ Building on this strategy, we evolved two additional PylRS strains from *M. mazei* and *Candidatus Methanomethylophilus alvus* (*Ca. M. alvus*) to incorporate BocK at the UAG codon and *N* ^ε^-acetyl-l-lysine (AcK) at the UAA codon in sfGFP1TAG134TAA (Figure 1B).^41^ To achieve this, we evolved the *Cma*PylRS–tRNA^Pyl^ pair through three rounds of random mutagenesis and screening, enhancing its catalytic efficiency. It was confirmed that *Cma*PylRS is orthogonal to *mm*tRNA^Pyl^, while *Mm*PylRS exhibits a high catalytic efficiency toward *Cma*tRNA^Pyl^. Introducing a C41AU mutation in *Cma*tRNA^Pyl^ blocked its recognition by *Mm*PylRS, preventing cross-reactivity.^41,42^ The R3-9 and R3-14 clones of *Cma*PylRS, carrying mutations Y16F/E161G/N182I and N57D/Y16F/E161G/N182I, respectively, displayed improved UAG suppression when it was paired with *Cma*tRNA^Pyl^_CUA_-C41AU and BocK was used as the substrate.^41^ These evolved *Cma*PylRS clones, combined with *Cma*tRNA^Pyl^-C41AU and *Mm*AcKRS1–*Mm*tRNA^Pyl^_UUA_, enabled the simultaneous incorporation of two different Lys-ncAA derivatives, BocK and AcK, at amber and ochre codons, respectively, into sfGFP. The multiplexed nature of this system offers significant potential for protein synthesis and functional studies, such as installing multiple Lys PTMs simultaneously, allowing for precise mimicry of complex biological states. This capability provides the potential to study PTM cross-talk to delineate complex functions involving intricate cross-interactions between different Lys PTMs. This multiplexed incorporation strategy not only expands the coding capacity for diverse Lys-ncAAs but also lays the biochemical foundation for downstream chemical modifications and functional studies, as discussed in the next section.

## 3 Advancing Lys-Based Bioorthogonal Chemistry and Chemical PTMs

### 3.1 Lys-ncAAs for Lys PTMs or Their Mimetics

Lys PTMs play a crucial role in epigenetic regulation and cellular signaling, yet precise chemical tools for installing site-specific Lys PTMs remain limited. Our research has systematically expanded the chemical biology toolkits for studying Lys PTMs with an emphasis on methylation and acylation by exploring novel Lys-ncAA encoding PylRS mutants and bioorthogonal chemistry. Lys methylation is a critical PTM that governs epigenetic regulation, transcriptional control, and signal transduction. This modification, occurring at mono-, di-, and trimethylation levels, serves as a molecular signal for the recruitment of chromatin-modifying complexes and transcription factors. Beyond histones, Lys methylation also regulates key non-chromatin proteins influencing cellular functions ranging from DNA repair to apoptosis.^13^ Despite its importance, understanding Lys methylation at a mechanistic level has been hindered by the difficulty of obtaining proteins with site-specific Lys methylation.^6^

To address this challenge, we developed a high-yielding chemical synthesis strategy for site-specific Lys methylation.^43^ Our approach utilizes genetically encoded Se-alkylselenocysteine (SeC), which undergoes a selective oxidative elimination reaction by H_2_O_2_, generating a dehydroalanine (Dha) intermediate with a conversion yield of >95% (Figure 3A). The Dha intermediate can then undergo a Michael addition reaction with thiol nucleophiles, achieving incorporation yields of 88% for monomethyllysine (Kme1), 85% for dimethyllysine (Kme2), and 83% for trimethyllysine (Kme3), respectively. Compared to conventional native chemical ligation or expressed protein ligation, our method offers higher efficiency, improved yields, and precise modification control, enabling the production of homogeneously modified histones at milligram-scale quantities. Limitations of this approach include its reliance on an analog instead of original Lys methylation as well as the loss of chirality at the Cα position.

**Figure 3:**
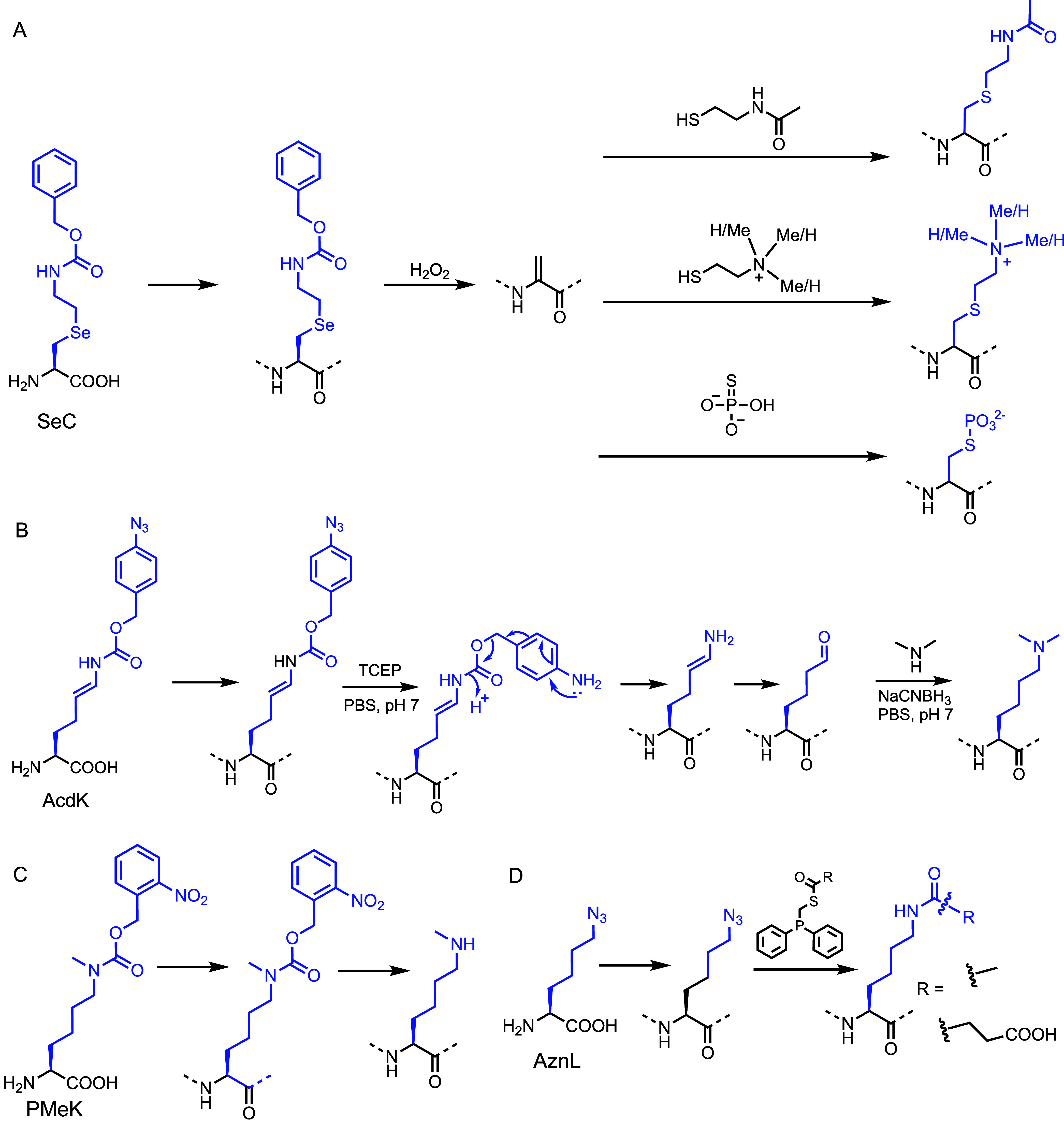
(A) Schematic illustration of SeC incorporation followed by oxidative elimination to generate Dha, enabling subsequent Michael addition. (B) Schematic illustration of AcdK incorporation followed by sequential Staudinger reduction, *para*-aminobenzyloxycarbonyl self-cleavage, enamine hydrolysis, and reductive amination to yield site-specific Kme2 within proteins. (C) Schematic illustration of PMeK incorporation followed by deprotection into MeK. (D) Genetic incorporation of AznL and site-specific Lys acylation via traceless Staudinger ligation with phosphinothioesters.

Furthermore, we developed a stereoselective and genetically encoded strategy for synthesizing proteins with site-specific Lys dimethylation.^1^ We genetically incorporated *N* ^ε^-(4-azidobenzoxycarbonyl)-δ,ε-dehydrolysine (AcdK) into proteins via amber codon suppression, enabling the precise installation of a Lys precursor (Figure 3B). We then selectively converted AcdK into allysine (AlK) under mild reducing conditions (5 mM TCEP, pH 7, 2 h), followed by stereoselective reductive amination with dimethylamine and 5 mM NaCNBH_3_ at pH 7 for 8 h, yielding proteins with Kme2. Our approach achieves >95% AlK recovery and >90% methylation efficiency, significantly improving selectivity over existing methylation strategies. This strategy ensures stereochemical control, avoids harsh conditions, and is applicable to large proteins such as histones and p53. The same approach was tested successfully on the installation of Kme1 and Lys alkylations as well. Compared to the aforementioned selenium-based chemical approach, this method leads to native forms of Lys PTMs and Cα chirality.

While reductive amination allowed for highly efficient and stereoselective installation of Kme2, it cannot be conducted in live systems. In 2010, we developed a photochemically controlled approach for the synthesis of proteins with site-specific Lys monomethylation. This is a light-responsive method to regulate Lys methylation with spatiotemporal precision.^44^ We incorporated *N* ^ε^-(*o*-nitrobenzyl)-methyl-l-lysine (photocaged MeK, PMeK) into proteins using an engineered PylRS–tRNA pair, achieving >95% incorporation efficiency (Figure 3C). Upon UV irradiation at 365 nm for 10 min, the *o*-nitrobenzyl group was efficiently removed, restoring Kme1 with a deprotection yield exceeding 92%.

Following our advances in site-specific Lys methylation, we extended our work to Lys acetylation. We developed a traceless Staudinger ligation strategy that enables precise and high-yielding site-specific Lys acetylation while preserving the native Lys architecture (Figure 3D).^8^ We first incorporated azidonorleucine (AznL) into proteins using an engineered PylRS–tRNA pair, achieving >95% incorporation efficiency in *E. coli* expression systems. AznL was converted to acetyllysine via traceless Staudinger ligation using 5 mM diphenylphosphinomethanethiol acetate. The phosphine reagent facilitated the azide-to-amine reduction, allowing direct acetyl group transfer without introducing unnatural linkers. To demonstrate the applicability of our approach, we synthesized homogeneously acetylated histone H3K4ac and ubiquitin K48ac, key regulators of chromatin structure and protein degradation, respectively.

### 3.2 Lys-ncAA-Based Bioorthogonal Chemistry

Building upon our work in genetic code expansion for Lys modifications, we genetically encoded an aliphatic keto-containing Lys derivative for protein bioorthogonal labeling and modification. While previous bioorthogonal methods relied on phenolic keto-containing amino acids such as *p*-acetylphenylalanine (*p*AcF), these suffered from low reactivity with hydrazide and alkoxyamine reagents due to electron-donating effects of the aromatic ring. To address this, we engineered the site-specific incorporation of 2-amino-8-oxononanoic acid (KetoK), an aliphatic keto-Lys analog, into proteins via an evolved PylRS/tRNA^Pyl^ pair, enabling highly efficient and selective labeling under mild conditions (Figure 4A).^45^ We evaluated the bioorthogonal reactivity of KetoK by labeling purified GFP-KetoK with 1 equiv of Texas Red hydrazide at pH 6.3, achieving quantitative labeling. The kinetic analysis of KetoK labeling with alkoxyamines revealed a rate constant (*k*_2_) of 3.1 M^–1^ s^–1^, which is significantly faster than that of *p*AcF (0.8 M^–1^ s^–1^).

**Figure 4:**
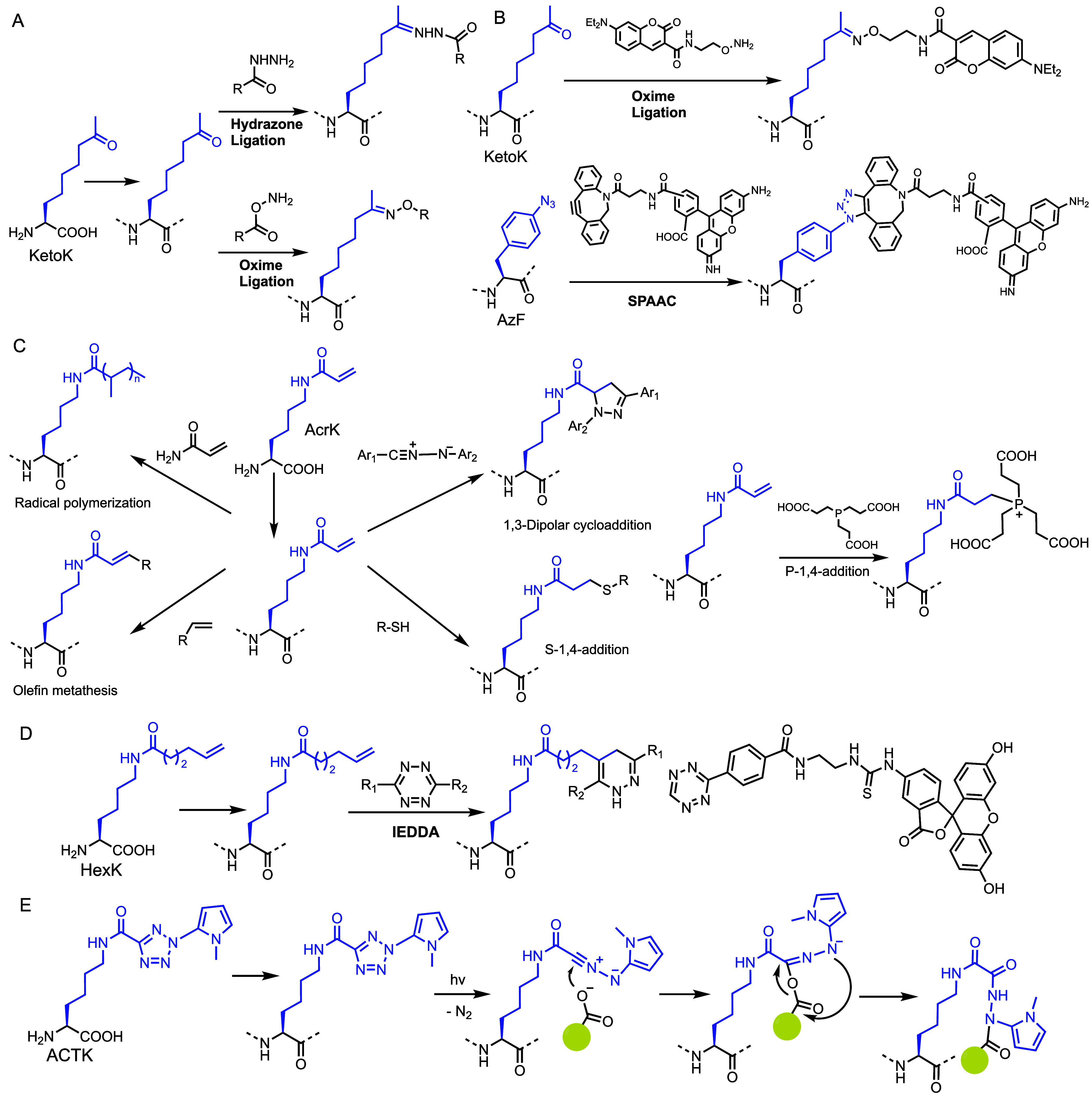
(A) Schematic illustration of KetoK incorporation and condensation reactions of the keto group in genetically encoded ketoK with hydrazides or alkoxyamines. (B) Bioorthogonal dual labeling: incorporated KetoK condenses with coumarin hydroxylamine via oxime formation while AzK reacts with Rhodamine DBCO via SPAAC click reaction. (C) Genetically incorporated AcrK as a versatile chemical handle enabling a range of on-protein reactions including radical polymerization, olefin metathesis, 1,3-dipolar cycloaddition, and S/P-1,4-conjugate addition. (D) HexK incorporation and reaction with tetrazine derivatives via IEDDA reaction. (E) ACTK incorporation and photo-cross-linking with a protein carboxylic group.

There are other dual labeling strategies for proteins that have been developed. They typically involve cysteine and often suffer from cysteine cross-reactivity, low efficiency, or reliance on metal catalysts, which can induce protein aggregation and oxidation. To overcome these limitations, we used two genetically encoded ncAAs, an aliphatic KetoK and a 4-azidophenylalanine (AzF), to direct two mutually orthogonal oxime formation or strain-promoted azide–alkyne cycloaddition (SPAAC) labeling reactions within the same protein (Figure 4B).^46^ The two reactions proceeded independently and without cross-reactivity, allowing us to install two distinct fluorophores for Förster resonance energy transfer (FRET) analysis, confirming protein conformational changes. This one-pot, catalyst-free approach provides a robust platform for dual-site-selective labeling, expanding chemical protein engineering applications in fluorescence imaging and protein dynamics studies.

Expanding our work in bioorthogonal Lys modifications, we developed a genetically encoded *N* ^ε^-acryloyl-l-lysine (AcrK) as a highly reactive handle for 1,3-dipolar cycloaddition, 1,4-conjugate addition, and radical polymerization (Figure 4C).^47^ Unlike ketones, alkynes, and azides, which have limited reactivity under physiological conditions, the electron-deficient olefin in AcrK offers broader reactivity and enhanced versatility. Using an engineered PylRS/tRNA_CUA_ pair, we incorporated AcrK into sfGFP expressed by *E. coli*, achieving an incorporation efficiency of over 95%. To explore its reactivity, we first examined 1,4-conjugate addition by reacting sfGFP-S2AcrK (50 μM) with β-mercaptoethanol (40 mM) at pH 8.8, 37 °C overnight, leading to near-complete thiol addition. PEGylation using methoxypoly­(ethylene glycol)­thiol (mPEGSH5k, 40 mM) under identical conditions resulted in 50% conversion. Next, we investigated radical polymerization, successfully embedding sfGFP-S2AcrK (110 μM) into a hydrogel through polymerization with 15% acrylamide and 0.5% bis-acrylamide. Furthermore, we integrated it with nitrilimine–alkene cycloaddition, enabling ultrarapid, catalyst-free protein labeling with a second-order rate constant exceeding 3.4 × 10^4^ M^–1^ s^–1^ at pH 10, making it among the fastest known bioorthogonal reactions.^48^ To further expand AcrK reactivity, we introduced phospha-Michael addition, leveraging TCEP as a nucleophile for rapid and selective modification.^49^ Unlike thiol-based Michael additions, which suffer from slow kinetics due to high p*K*_a_ and poor nucleophilicity, phosphines provide stronger nucleophilicity due to their polarizable lone pair, leading to significantly faster conjugate addition. The second-order rate constant for TCEP addition to AcrK (0.067 M^–1^ s^–1^ at pH 7.4) far exceeds that of thiols (≤0.01 M^–1^ s^–1^). Reacting sfGFP-AcrK (50 μM) with 2 mM TCEP at pH 8.8, 37 °C for 1 h resulted in >90% conversion. This enhanced reactivity broadens the utility of AcrK for efficient bioconjugation in live cells. Beyond small-molecule conjugation, we leveraged arylamide-mediated intramolecular cyclization to enable an efficient and cysteine selective 1,4-conjugate addition reaction for *in situ* peptide macrocyclization displayed on phages, without requiring additional reagents and extra reaction procedures like disulfide or linker-based cyclization.^50^ Using a randomized 6-mer phage-displayed library, we enriched high-affinity cyclic peptide binders against TEV protease and HDAC8, which exhibited 4–6-fold stronger binding than their linear counterparts, respectively. This method establishes a genetically encoded phage-displayed cyclic peptide platform, expanding AcrK’s utility for drug discovery.

To expand our work in bioorthogonal Lys modifications, we explored the potential of genetically encoded unstrained alkene-functionalized Lys derivatives for inverse electron-demand Diels–Alder cycloaddition (IEDDA) (Figure 4D).^51^ Traditional IEDDA reactions primarily rely on strained cyclic olefins, such as norbornene or *trans*-cyclooctene, which exhibit high reactivity but suffer from synthetic complexity and limited stability in biological environments. To overcome these challenges, using an engineered PylRS/tRNA^Pyl^_CUA_ pair, we successfully incorporated unstrained alkene-bearing *N* ^ε^-hex-5-enoyl-l-lysine (HexK) into sfGFP under optimized conditions (1 mM IPTG, 5 mM HexK, 0.2% arabinose). Kinetic studies of HexK with tetrazine fluorophores in PBS at room temperature revealed a second-order rate constant of 0.016 M^–1^ s^–1^, comparable to that of SPAAC (0.0565 M^–1^ s^–1^). We achieved efficient Lys-based tetrazine ligation using unstrained olefins, labeling HexK-incorporated sfGFP with 50% efficiency *in vitro* and enabling selective fluorescent tagging of OmpX in *E. coli* without toxicity. This establishes unstrained alkene-functionalized Lys as a biocompatible alternative to strained systems for *in vivo* bioorthogonal labeling and protein conjugation.

Traditional photo-cross-linkers such as diazirines, benzophenones, and aryl azides generate highly reactive radicals or nitrenes, leading to nonselective cross-linking with nearby residues. To improve selectivity, we collaborated with Qing Lin’s lab to develop *N* ^ε^-(2-aryl-5-carboxytetrazolyl)-l-lysine (ACTK), a Lys analog bearing 2-aryl-5-carboxytetrazole (Figure 4E).^52^ Upon 302 nm UV irradiation, ACTK forms a nitrile imine that cross-links nearby nucleophiles. Incorporation into sfGFP and glutathione-*S*-transferase (GST) via engineered PylRS/tRNA^Pyl^ enabled site-specific photo-cross-linking with ∼53% yield at E52 after 5 min. Compared with diazirines, ACTK offers enhanced efficiency and selectivity without side reactions, making it a valuable tool for probing protein interactions.

Together, our work in GEK bioorthogonal modifications has significantly expanded the chemical biology toolkits for site-selective protein labeling, conjugation, and cross-linking. By engineering Lys derivatives with reactive keto, azide, acrylamide, alkene, and photo-cross-linking groups, we have developed a versatile and orthogonal platform for precise biomolecular functionalization under physiological conditions. These approaches enable efficient fluorescence labeling, covalent protein interaction mapping, peptide cyclization, and therapeutic modifications, complementing conventional protein engineering strategies, highlighting the power of GEK modifications in chemical biology and biomedical research.

## 4 Structural and Functional Study on Lys PTMs

The most reported PTM is the acylation of the ε-amino group of Lys residues. The degree of acylation affects the state of nucleosome aggregation and therefore of gene expression. Acylated histones form less highly condensed hetero chromatin than histones without acylation, resulting in disaggregation of the nucleosomes. Also, acylation neutralizes the positive charge on the free amino group of Lys residues and increases the hydrophobicity. The decreased electrostatic interactions provide opportunities for chromatin remodeling, which means nucleosomes can slide along a DNA molecule, exposing sequences for transcription. Acylated histones also recruit acyllysine epigenetic readers which are essential for epigenetic processes.

To study the interactions between acylated histones and epigenetic related proteins, one method is to incorporate an ε-amino acylated Lys into histones, then reconstitute acyllysine incorporated nucleosomes *in vitro*, enabling structural analysis. Also, ε-amino acylated Lys with a chemically active acyl group can be applied to bioorthogonal reactions for labeling proteins. Our group together with collaborators have successfully incorporated several different Lys-ncAA variants into histones to facilitate structural insights for epigenetics.

### 4.1 *N* ^ε^-Acetyl-l-lysine (AcK)

Sirtuins are class III histone deacetylases. Seven sirtuins have been identified in humans, namely, SIRT1–7. To completely understand the overall character of SIRT1 and SIRT2 on the nucleosome, we proposed incorporation of *N* ^ε^-acetyl-l-lysine (AcK) (Figure 5A) into histone H3 with an ELISA-based rapid throughput assay approach to identify which lysine residues on histone H3 are targeted by SIRT1 and SIRT2.^53^ We incorporated AcK into several lysine residue sites of histone H3. Then together with other histone proteins and DNA, the nucleosome was assembled *in vitro*. The removal of incorporated acetyl Lys by SIRT1 and SIRT2 can be detected by the anti-Kac antibody using ELISA (Figure 5B). We found that SIRT1 and SIRT2 exhibited heightened enzyme activities toward nucleosome substrates compared with histone H3 peptides. Moreover, almost every acetylation installed in histone H3 was removed by SIRT1 and SIRT2, indicating little site specificity of the SIRT1 and SIRT2 reactivity *in vitro*. However, *in vivo* studies reveal SIRT1 and SIRT2 have higher preferences toward some Lys sites. The substrate specificity may be dependent on binding partner and the cellular context where they are needed.

**Figure 5:**
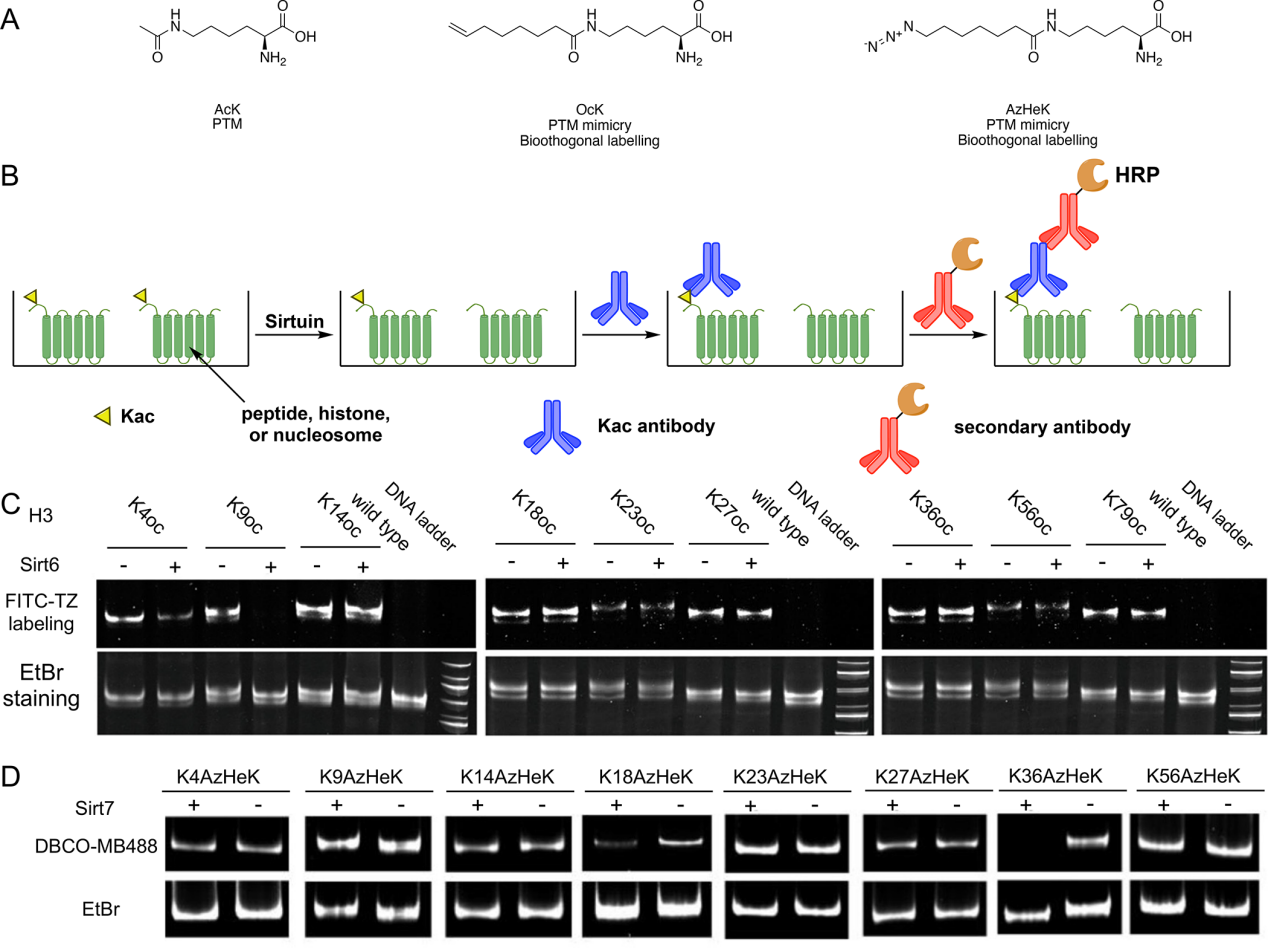
(A) Chemical structure of *N* ^ε^-amino acylated Lys incorporated in our study. (B) ELISA system for site recognition assays of sirtuin enzymes. (C) Sirt6 activities on oc-nucleosomes. (D) SIRT7-catalyzed deacylation activities on eight acyl-nucleosome substrates. The AzHeK residue which could be recognized by Sirt7 was removed and thus was incapable of conjugating DBCO-MB488 dye, resulting lack of fluorescence.

### 4.2 *N* ^ε^-(7-Octenoyl)-l-lysine (OcK)

SIRT6 is also a HDAC identified in humans as a key regulator of mammalian genome stability, metabolism, and life span. Compared with acetylated Lys residues, SIRT6 exhibits an enhanced activity toward the removal of long fatty acyl chains from lysine.^54^ We incorporated *N* ^ε^-(7-octenoyl)-l-lysine (OcK) (Figure 5A) into different Lys residue sites of histone H3. Compared with *N* ^ε^-decanoyl-l-lysine (DeK), which is a common fatty acylated Lys residue recognized by SIRT6, OcK exhibits high structural similarity due to the similarity between the terminal olefin and the ethyl group. We then treated the assembled nucleosome with incorporated OcK and labeled by tetrazine conjugated dye with SIRT6. We found that not only K9 but also K18 and K27 are targets for SIRT6 deacylation (Figure 5C). In our study, we clearly observed that K56 is not a deacetylation site for SIRT6, and some developed antibodies for H3K56 showed strong cross-reactivity to recognize H3K9ac, leading to likely misinterpretation of some biological data.

### 4.3 *N* ^ε^-(7-Azidoheptanoyl)-l-lysine (AzHeK)

SIRT7 is the least studied sirtuin and is believed to have effects on cellular homeostasis, oncogenic potential, and cellular aging pathways.^55^ It has been confirmed that SIRT7 is able to recognize and deacetylate histone H3K18.^12^ However, molecular details of interactions of SIRT7 with nucleosomes for deacetylation have not been investigated. We incorporated *N* ^ε^-(7-azidoheptanoyl)-l-lysine (AzHeK) (Figure 5A) into K4, K9, K14, K18, K23, K27, K36, and K56 sites of histone.^2^ AzHeK is expected to mimic *N* ^ε^-decanoyl-l-lysine (DeK), which will allow efficient recognition by SIRT7 for deacylation. AzHeK has an azide group which allows click reaction with a DBCO dye for rapid labeling. As shown in Figure 5D, SIRT7 was able to remove acylation from H3K18 and H3K36 but did not show reactivity toward other lysine residues. We further investigated whether free DNA had effects on SIRT7 catalyzed nucleosome deacylation. The H3K36AzHeK-containing nucleosome was treated with different concentrations of DNA. As shown in Figure 5C, free DNA resulted in decreased removal of the acyl group, indicating an inhibition of SIRT7 deacylation activity, which results from the electrostatic interactions between positively charged SIRT7 and negatively charged DNA. DNA serves as a mediator between SIRT7 and an acyl histone for deacylation. The biochemical fractionation and chromatin immunoprecipitation studies also show the pivotal role of SIRT7 in maintaining a low H3K36ac level, which may contribute to rDNA heterochromatin silencing and stability, active transcriptional elongation, or DNA repair.

## 5 Lys-ncAA-Assisted Drug Discovery

Based on the structural and mechanistic understanding of Lys residues in epigenetic regulation as dynamic sites for PTMs, we aim to leverage substrate specificity and Lys reactivity to guide the development of selective ligands. **P**hage-assisted **A**ctive Site-**D**irected **L**igand **E**volution (PADLE) is a versatile platform we developed for discovering selective ligands targeting Lys-modifying and Lys-recognizing proteins.^3,4,56^ By incorporating Lys-ncAA into phage-displayed peptide libraries via amber suppression, PADLE allows the precise anchoring of ligands to catalytically or structurally critical Lys sites, thereby enabling active-site-guided enrichment via PADLE biopanning (Figure 6A).

**Figure 6:**
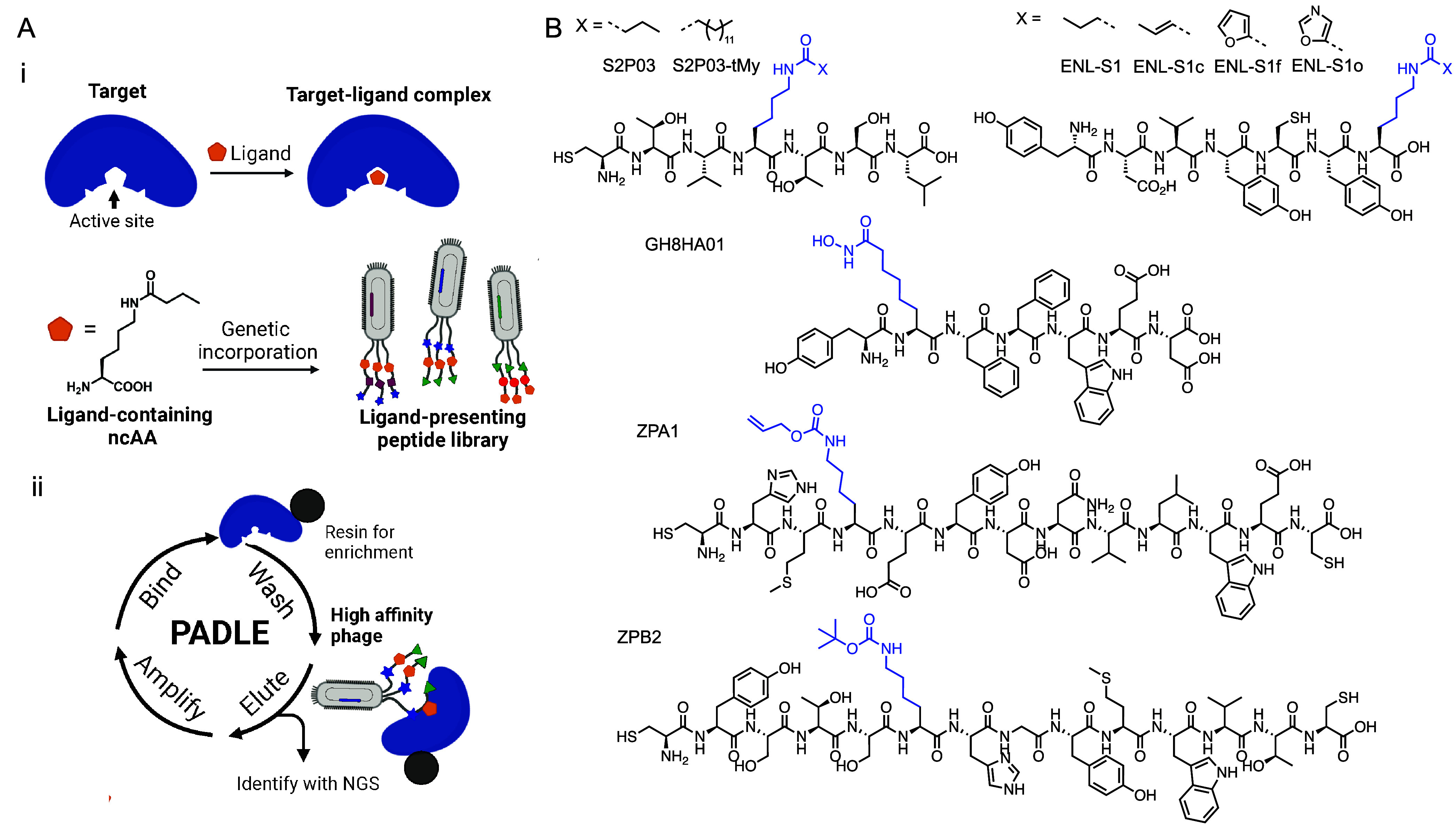
(A) Schematic diagram of PADLE. (i) ncAAs such as *N* ^ε^-butyryl-l-lysine are genetically incorporated into phage-displayed peptide libraries to anchor ligands at enzymatic active sites. (ii) PADLE biopanning procedure. (B) Chemical structures of PADLE-evolved peptide ligands, each containing a Lys-ncAA (highlighted in blue).

The enzymes including HDACs, sirtuins, and YEATS domain proteins, are significant therapeutic targets in cancer, neurodegeneration, and immune disorders.^16,55,57^ However, selectively targeting these proteins remains difficult due to conserved catalytic domains and dynamic modification states. PADLE circumvents these challenges by leveraging the intrinsic substrate specificity and reactivity of Lys-binding pockets through structure-guided Lys-ncAA incorporation. We first demonstrated this strategy using BuK to evolve selective inhibitors for class III deacetylase SIRT2, whose natural substrate preference favors butyrylated Lys. Screening BuK-containing libraries yielded high-affinity ligands (Figure 6B) such as S2P03 (*K*_d_ = 49 nM), with further optimization producing S2P03–tMy (*N* ^ε^-thiomyristoyl-l-lysine (tMyK) (IC_50_ = 10 nM).^3^ Structural analysis confirmed that BuK mediates hydrophobic interactions with nonconserved residues (F235, L239), conferring isoform specificity. We then applied the same principle to the ENL YEATS domain, a reader protein that selectively recognizes BuK and *N* ^ε^-crotonyl-l-lysine (CrK) marks. PADLE screening identified ENL-S1 (*K*_d_ = 36.3 nM, IC_50_ = 63 nM) (Figure 6B), which leveraged π–π stacking and hydrogen bonding via BuK to achieve selectivity over the homologous AF9 YEATS.^4^ Substitution with *N* ^ε^-5-oxazole-carbonyl-l-lysine (OxaK) yielded ENL-S1o (*K*_d_ = 2.0 nM). A truncated version, ENL-S1f, inhibited ENL-driven transcription (IC_50_ = 6.4 μM) and suppressed leukemia proliferation, validating PADLE for reader protein inhibition. The strategy was next extended to the Zn^2+^-dependent deacetylase HDAC8, where canonical inhibitors struggle with isoform selectivity. Using Aoda (an AcK isostere) and PADLE selection, we identified GH8HA01 (IC_50_ = 0.85 nM), the first subnanomolar HDAC8 inhibitor (Figure 6B).^56^ Beyond enzymatic targets, PADLE was adapted to modulate ligand–receptor interactions, using BocK and AllocK to screen against ZNRF3, a membrane-bound E3 ligase involved in Wnt signaling.^14^ Optimized peptides ZPB2 and ZPA1 showed *K*_d_ values of 1.6 and 5.9 μM, respectively (Figure 6B). These Lys-ncAA variants enabled interactions not accessible with canonical Lys, with steric and electrostatic features critical for selective binding. Together, these studies establish PADLE as a generalizable framework for the discovery of potent and selective ligands across epigenetic enzymes, reader domains, and membrane receptors, driven by strategic Lys-ncAA incorporation to match target binding site properties.

## 6 Conclusions

In conclusion, GEK chemistry has become a pivotal tool in precise protein engineering and therapeutic innovations, enabling modifications beyond its natural post-translational roles. Our advances in bioorthogonal Lys chemistry now allow site-specific functionalization with exceptional selectivity, facilitating precise protein labeling and regulation. Meanwhile, the GEK derivatives, enabled by our evolved PylRS–tRNA^Pyl^ pairs, have expanded the Lys modification scope by introducing validated photoactivatable, electrophilic, and structurally diverse analogs into proteins with high fidelity. These innovations have facilitated epigenetics, structural biology, and drug discovery, where GEK modifications enable precise biomolecular interactions and artificial PTM engineering. Moving forward, the integration of genetic code expansion, Lys relevant bioorthogonal reactions, and synthetic PTM control will further drive advances in chemical biology, peptide/protein therapeutics, and personalized medicine such as lysine-directed covalent probes, Lys-ncAA-based PROTAC design, and programmable chromatin modifiers, by enabling Lys-specific installation of functional handles or PTM mimetics. While our current efforts focus on *E. coli* and cell-based systems, successful *in vivo* delivery of engineered tRNA–synthetase pairs and maintenance of substrate bioavailability and orthogonality in complex physiological environments could enable future extension of GEK strategies to whole-animal models.
